# Linking gene expression to phenotypes via pathway information

**DOI:** 10.1186/s13326-015-0013-5

**Published:** 2015-04-11

**Authors:** Irene Papatheodorou, Anika Oellrich, Damian Smedley

**Affiliations:** Mouse Developmental Genetics, Wellcome Trust Sanger Institute, Wellcome Trust Genome Campus, CB1 10SA, Hinxton, UK

**Keywords:** Phenotypes, Pathways, Gene expression, Ontologies

## Abstract

Establishing robust links among gene expression, pathways and phenotypes is critical for understanding diseases and developing treatments. In recent years there have been many efforts to develop the computational means to traverse from genes to gene expression, model pathways and classify phenotypes. Numerous ontologies and other controlled vocabularies have been developed, as well as computational methods to combine and mine these data sets and establish connections. Here we discuss these efforts and identify areas of future work that could lead to a better integration of genes, pathways and phenotypes to provide insights into the mechanisms under which gene mutations affect expression and pathways and how these effects are manifested onto the phenotype.

**Electronic supplementary material:**

The online version of this article (doi:10.1186/s13326-015-0013-5) contains supplementary material, which is available to authorized users.

## Introduction

A fundamental aspect of disease research involves the understanding of biological processes that underpin observed phenotypes. In order to achieve this level of understanding, diseases need to be described as collections of measured phenotypes and these phenotypes need to be analysed in relation to their genetic causes and genomic effects and linked with information on molecular interactions. One consequence of these efforts could be the ability to produce predictive models of phenotypes from genomic profiles with the aim of describing diseases more accurately. Such models will be helpful in understanding the genetic basis and molecular mechanisms leading to complex or rare developmental diseases and the process of ageing, as well as the characterisation and progression of cancer types. In particular, models built from model organism datasets can be translated into insights on humans in areas such as disease gene identification and drug target testing.

Methods for assigning genotypes to phenotypes have been developed and used intensively [1-3]. These methods include genome wide association studies (GWAS) that are applied to identify causative genotypes for various conditions and phenotypes. For example, a case-controlled genome wide association study identified five loci to be associated with the susceptibility of osteoarthritis [4]. However, the identification of loci (and with that the genotype) still leaves a gap as to what molecular mechanisms are at play to yield the observed phenotype. As a consequence, GWAS studies are usually followed by functional experiments, trying to unravel the biological mechanisms that could influence the phenotype given the identified genotype.

A functional follow-up experiment to fill the gap between genotype and phenotype is the assessment of expression levels of genes in the vicinity of the identified GWAS loci in one or more tissues. In the study concerning osteoarthritis [4], the authors investigated further the gene expression and the protein expression of associated genes using RT-PCR (genes) and immunohistochemical staining. Through these functional studies, they identified high levels of nucleostemin (encoded by the GNL3 gene) in osteoarthitis patients.

A potential next step in connecting the identified genotype with the phenotype is to establish a link between the expression of genes and the observed phenotypes, which has been attempted by numerous studies [5-7]. A recent example involves the characterisation of phenotypes in yeast using high-throughput transcriptomic analyses [5]. Data classification methods have been used extensively to characterise healthy or diseased tissue [6] from the context of gene expression.

In the examples of the GWAS and high-throughput gene expression studies, although the genetic and genomic outcomes of the disease can be associated with phenotypes, the biological events leading to the phenotype at the systems level are not discovered. Signalling and metabolic pathway analyses can inform on the specific mechanisms of the genetic causes of the phenotypes. Recent work by Harper *et al.* [8] presents a method for augmenting pathway data with phenotypes from high-throughput genetic screens in bacteria in order to discover causative genes.

**Figure 1:**
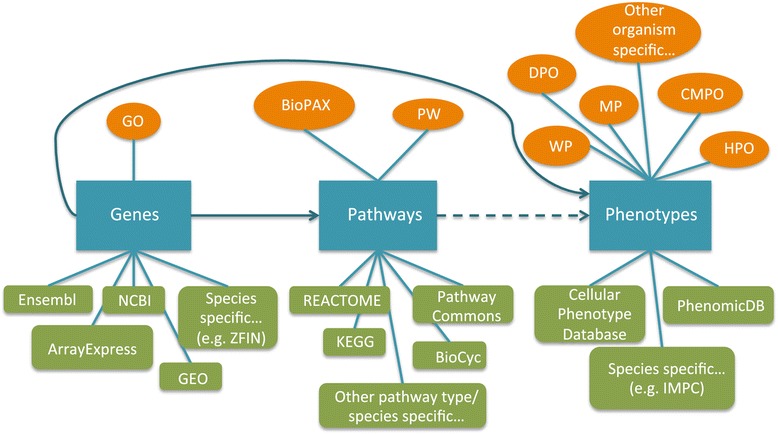
**Databases and ontologies for information on genes, pathways and phenotypes.** The diagram shows the information flow from genes to phenotypes via pathways. There are a large number of databases storing gene expression and other genomic data, with most of them species specific that include links to a phenotype ontology term. In addition, there is a large number of phenotype ontologies that are not organism specific, such as a mammalian phenotype ontology and the cellular phenotype ontology (CMPO). There exist a few databases providing genotype to phenotype links, although most of this information is covered by species specific genomic databases. There are many small-scale species specific or pathway-type specific databases and a few large general pathway databases (KEGG, Pathway Commons, REACTOME). Pathway ontologies exist but are not widely used yet. Although in general there are good links among genes and pathways and genes and phenotypes, associations between pathways and phenotypes are lacking

To date, many databases (e.g. [9-11]) and ontologies (e.g. [12-14]) have been developed to describe genes, pathways and phenotypes across different species (see Figure 1 and Additional file 1). However, the semantic integration of these resources needed to computationally analyse the experimental set-up described above, is still at its infancy. This hinders the development of generalised data analysis methods that combine genes, gene expression, pathways and phenotypes. Here, we identified three areas of research that need to be further developed in order to facilitate computational prediction of the biological mechanisms that link genotypes to phenotypes: (i) the ontological characterisation of phenotypes, (ii) linking gene expression and phenotypes and (iii) linking pathways and phenotypes. We describe the current state-of-the-art for each of the three areas in the following sections individually and highlight potential future challenges where identified.

## Ontological characterisation of phenotypes

**Figure 2:**
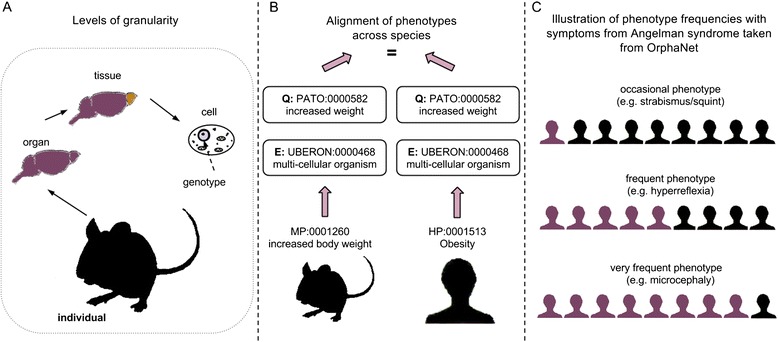
**Three challenges when representing and comparing phenotypes.** The diagram illustrates the three challenges that need to be overcome in order to link gene expression and phenotypes using pathways: **(A)** the integration across the different levels of complexity within an organism, **(B)** the integration across species, and **(C)** the frequencies of occurrences of phenotypes (quantification) – purple colour represents individuals possessing phenotype of interest (examples in parentheses taken from Angelman syndrome in OrphaNet)

Due to the availability of phenotype data from several model organisms (see Figure 1, e.g., phenotypes from the International Mouse Phenotyping Consortium [15]), options are not limited to human systems but may include data from several different species. Furthermore, data obtained through different experiments and stored in different data resources can vary in the detail the information is represented with [16]. As a consequence, three major aspects of data integration need to be addressed: (i) the integration across the different levels of complexity within an organism, (ii) the integration across species, and (iii) the frequencies of occurrences of phenotypes (quantification). These three aspects are further illustrated in Figure 2. To facilitate data integration, numerous ontologies have been developed that define the meaning of biological concepts, such as the Gene Ontology (GO) [13].

### Integration across different levels of organismal complexity

Phenotypes span different levels of complexity and range from a molecular level to the entire organism, such as the cellular level, the tissue level or the organ level (see Figure 2(A)) [17]. Existing biomedical ontologies cover several levels of complexity, e.g., ontologies that represent gene function (GO) as well as tissue information (e.g. BRaunschweig ENzyme DAtabase (BRENDA) tissue ontology (BTO) [14]) or organism level (e.g. the Mammalian Phenotype Ontology (MP) [12] or the Human Phenotype Ontology (HPO) [18]). In order to facilitate reasoning over the different levels of complexity needed to describe an individual with ontologies, the ontologies have to be aligned and mapped to one another. While mapping efforts are ongoing to align ontologies across species covering the same level of complexity, e.g., the alignment of anatomy as provided by UBERON [19], the seamless integration of ontologies across the different levels of complexity is still ongoing work.

### Integration across species

In order to computationally compare phenotypes across different species, the existing phenotype data needs to be semantically annotated in a way that would facilitate the comparison. Traditionally, model organisms as well as human data were semantically represented using pre-composed phenotype ontologies. In a pre-composed phenotype ontology such as MP or HPO, one concept corresponds to one phenotype and can directly be used for annotation. However, a comparison is only possible as long as the same pre-composed ontology is used for annotation.

To overcome this limitation of pre-composed phenotype ontologies, post-composed phenotype representations have been suggested. One approach that is broadly used, for example to post-compose MP and HPO and represent zebrafish mutants in the Zebrafish Model Organism database [20], is the description of phenotypes using Entity-Quality (EQ) statements. Entity-Quality (EQ) statements enable the composition of phenotypes using species-independent ontologies [21], e.g. GO (for the representation of processes) or UBERON (a cross-species anatomy ontology). While some of the statements have been generated and verified automatically [22], manual verification is still needed to ensure the correct representation. How species can be compared based on pre- and post-composed phenotype annotations is illustrated in Figure 2(B).

The applicability of the generated EQ statements is demonstrated by their usage in a variety of projects, which e.g., predict the involvement of genes in diseases and pathological processes [2,3] and gene function [23]. Despite the successful applications of pre- and post-composed phenotype annotations, the harmonised application of phenotypes in conjunction with gene, expression and pathway data is still very limited.

### Frequencies of occurring phenotypes

The quantification of phenotype data is beginning to become available: databases such as OrphaNet [24] describe disease phenotypes with additional quantifiers, e.g., the phenotype *dwarfism* (OrphaNet clinical sign id: 53350) is *very frequent* in patients with a 12q14 micro deletion syndrome (OrphaNet disorder id: 12544) or the phenotype *strabismus* (OrphaNet clinical sign id: 5870) is occurring *occasionally* in patients with Angelman syndrome (OrphaNet disorder id: 90). OrphaNet assigns phenotype annotations and frequency information represented with an OrphaNet-specific vocabulary (see Figure 2(C)).

A similar strategy has been applied to annotate human genetic disorders described in the Online Mendelian Inheritance in Man (OMIM) database [9]. Each disorder is described using concepts of the HPO and, optionally, frequency information can be added to each of the assigned phenotype annotation [25]. Despite great efforts, frequency information is not available for all the annotations assigned and only available via the download file.

While clinical databases already work on the inclusion of quantified phenotype data, model organism databases lag behind by not providing this information. Thus, quantified phenotype information cannot yet be used for cross-species data analysis and computational modelling.

## Linking gene expression to phenotypes

The ease of obtaining whole genome expression datasets has enabled more thorough classification of phenotypes associated with the expression of sets of genes [6]. A large number of studies attempt to identify groups of genes whose expression is responsible for a particular phenotype, such as disease or tissue morphology [26]. More complex experimental designs attempt to associate the phenotype with dynamic or systems views of gene expression [27]. The techniques used to link the phenotype to causative patterns of gene expression largely depend on the experimental design and the technology used to profile gene expression.

### Gene expression signatures

In order to characterise a phenotype in terms of gene expression, most studies attempt to identify the minimum number of genes whose expression patterns determine the phenotype in question. This group of genes is referred to as a “gene signature” in the literature and once defined and validated has important practical applications to disease diagnosis and prognosis, as well as the discovery of new therapies. In cancer genomics, for example, classifications of tumour types from high-throughput gene expression and/or copy number profiles have helped unravel the complexity of different cancer types and have led to a better understanding of cancer progression and the identification of new diagnostic biomarkers. For example, Marisa *et al.* [6] produced a transcriptome-based classification of 566 colon cancer samples to discover six different molecular subtypes of the disease, that associated with distinct clinicopathological characteristics and corresponded to different relapse times. Aravinthan *et al.* [28] defined a signature of 40 genes that appear upregulated in hepatocyte senescence as opposed to controls and then validated this by finding enrichment of these genes in public data sets representing liver conditions such as steatohepatitis, alcohol-related hepatitis and HCV-related cirrhosis [28].

Given enough data sets, existing data mining methods can assign patterns of gene expression to the phenotypes under study. Although the linkage of gene expression signatures to phenotype associations is an important step in determining the causal link between genotypes and phenotypes, it is still difficult to establish the underlying biological mechanism from gene expression data sets alone.

### Complex experimental designs

More complex experimental designs are used in order to refine the mechanisms under which gene expression can lead to a certain phenotype. Here, the experimental design attempts to address issues such as the influence of environmental factors, time and interplay between tissues. An individual study example comes from the work by Äijö *et al.* [27] where statistical modelling based on Gaussian processes is used to analyse the differentiation of human Th17 cells. The authors expose CD4+ T cells to two different types of ligands and record the gene expression using RNA-seq over five different time-points. The analysis can describe the dynamics and provide insight into the kinetics of gene expression that lead to the different outcomes of T cell activation depending on the ligands used.

Tissue-specific and temporal based gene expression with matching phenotype measurements could be identified by appropriate experimental controls. However, these are often absent or impractical to implement in large-scale phenotyping assays or in cases of meta-analyses from already available data sets [7]. Generally, for more complex experimental designs to be more case-specific, custom-made computational solutions are usually required in order to analyse the gene expression data according to the different variables. Efforts are being made to generalise these tools and resources so that analysis of complex designs can be made easier. Examples include software for the analysis of time-series data sets. The DyNB tools suite in [27] and NextmaSigPro [29] are examples of software tools that enable analysis of time-series data sets.

Efforts have also been made to tackle the complexities of tissue specific gene expression in whole organism gene expression data sets by developing resources such as tissue specific gene expression atlases [30-32]. These data sets can be used as benchmarks to explore experiments on whole organisms. Small organisms such as Drosophila Melanogaster are difficult to dissect on a large scale and sometimes tissue specific expression must be inferred from whole body profiles, rather than directly measured. Innocenti *et al.* [33] extracted tissue specific genes from whole fly gene expression by use of FlyAtlas [30,34]. FlyAtlas is a database that holds information on genes expressed in 25 adult fly tissues originally obtained by tissue specific microarray profiling in wild type flies.

Gene expression analyses are very useful in identifying groups of genes that could characterise a phenotype. Although they do not provide much detail on the specific mechanisms under which the original stress or mutation leads to the observed phenotype, they can be used as a starting point for subsequent analyses that can narrow down candidate pathways and processes and generate hypotheses for more detailed experiments that can eventually shed light on the exact causes of the phenotype.

## Linking pathways to phenotypes

Deriving the underlying mechanism of the phenotype, given the initial mutations and/or resulting gene expression, involves the integration of knowledge on protein interactions and pathways [35]. There are different types of pathway analyses frequently used depending on the nature of the pathways: protein-protein interactions; gene-regulatory pathways; quantitative reaction modelling that includes metabolic, pharmacokinetic modelling. Methods for analysing these types of pathways have been previously reviewed in [35,36]. Linking these types of analyses with gene expression depends on whether there are already candidate pathways of interest and what is their degree of annotation. It also depends on the hypotheses of the studies investigated and whether they involve a small and specific pathway where knowledge of quantitative reactions matter and are available or whether they involve a large integrational study where broadness of pathway connections are important, usually at a cost of using detailed quantitative information on the kinetics of the interactions involved.

### Pathway models

Data for quantitative pathway analyses usually come from direct protein level measurements, therefore enabling the use of computational simulations for the formulation of predictive hypotheses that can subsequently be tested experimentally. Such approaches have the potential to produce predictive mathematical models describing the underlying mechanisms at high-levels of detail [37]. Panetta *et al.* [38] study the variations of methotrexate accumulation in cells of acute lymphoblastic leukemia patients using pharmacokinetic and pharmacodynamic models. By employing these methods they characterise how perturbations in the folate pathway, target of methotrexate, vary across the tumour subtypes (phenotypes) and how they relate to genetic variation and gene expression.

Quantitative pathway modelling methods are not easy to implement on a large scale and are mostly useful when there is already substantial knowledge of the biological process involved. In cases where the underlying biological process is unknown or poorly defined, high-throughput protein interaction data or high-level pathway information from pathway databases can help disentangle the mechanisms that are responsible for or induced by the observed gene expression. Boolean logic and other logic-based approaches, such as [39,40], have been used successfully for qualitative pathway analyses, to generate hypotheses that link gene expression, pathways and phenotypes.

### Knowledge integration

Pathway databases such as REACTOME [10] or KEGG [41] contain a wide range of developmental, signalling, metabolic, as well as disease pathways. These are well-linked to other resources, such as Ensembl [11] and Uniprot [42], for better integration with gene and protein information. Currently they support pathway enrichment analyses for a set of interesting genes or proteins and provide tools for visualising the pathways in the context of these interesting molecules. In addition, REACTOME provides ontological links between pathways, therefore allowing the exploration of interactions and relationships across different pathways. Often these pathway resources do not contain exactly the same pathways and in order to enable more comprehensive analyses, their data sets need to be merged. Resources such as BioSystems [43] attempt to collect and disseminate all available pathways from the available databases. However, due to lack of a widely used controlled vocabulary describing the available pathways, such attempts fail to fully semantically integrate data from different pathway databases. There has been significant progress in developing ontologies and standard formats for descriptions of pathway components and reactions (SBO, SBML, [44]). However, these have mainly been focused on describing the mathematical interactions within pathways in order to enable simulations. Therefore, they have not been widely adopted by all pathway databases in order to enable more effective integration.

Further work also needs to focus on linking the different levels of information, protein levels, gene expression and metabolic and signalling pathways into computational models that can handle qualitative and quantitative pathway parameters. Integrating different kinds of data sets from different species to solve a single, common biological process is an invaluable step in pathway analyses, but remains a difficult task. Advances in text-mining methods, as well as more accurate orthologous relationships between the genes of different species will help overcome these problems.

A major remaining problem in the linkage of genes and their expression signatures to pathways to phenotypes is the limited knowledge of the mapping between pathways and phenotypes. This is a difficult task mainly due to the lack of appropriate data sets that would enable the inference of such connections on the large scale. However, high-throughput phenotyping projects such as the International Mouse Phenotyping Consortium [15] have the potential to provide sufficient data sets for the inference of such links.

Finally, recent efforts on multi-scale models of organs attempt to bridge the gap between molecular pathways and physiology through projects such as the Virtual Physiological Human [45] and the Virtual Liver, a collaborative effort to produce a physiological model of the liver that interacts with pathways and other molecular component in order to support simulations and the understanding of the liver function in health and disease [46]. Such efforts are still in their initial steps but have the potential to facilitate a better understanding of the relationship between genes and phenotypes.

## Conclusions

High-resolution gene expression data sets are providing more insight into the functional consequences of the genotype as well as clues into the mechanisms that might control the phenotype. At the same time, research utilising pathway analysis and data integration has been increasingly important in explaining the biological mechanisms under which genotypes (and gene expression) influence phenotypes. Some form of pathway analysis is routinely part of gene expression studies. However, this is hindered by the lack of detailed pathway maps and quantitative information on the reactions. From the perspective of phenotype characterisation, the development of different types of ontologies and links between them is increasingly improving the integration of gene, tissue, anatomical and disease data sets within and between species. These improvements are creating the basis for more detailed associations between genes, pathways and phenotypes in the future.

## Additional file

Additional file 1
**Table detailing all databases and ontologies that appear in the text.**
